# Regulation of Kv11.1 Isoform Expression by Polyadenylate Binding Protein Nuclear 1

**DOI:** 10.3390/ijms22020863

**Published:** 2021-01-16

**Authors:** Matthew R. Stump, Rachel T. Nguyen, Rachel H. Drgastin, Delaney Search, Qiuming Gong, Zhengfeng Zhou

**Affiliations:** 1 Department of Biological and Molecular Sciences, George Fox University, Newberg, OR 97132, USA; rnguyen15@georgefox.edu (R.T.N.); rdrgastin16@georgefox.edu (R.H.D.); lsearch17@georgefox.edu (D.S.); 2 Knight Cardiovascular Institute, Oregon Health & Science University, Portland, OR 97239, USA; gongq@ohsu.edu (Q.G.); zhouzh@ohsu.edu (Z.Z.)

**Keywords:** alternative polyadenylation, hERG, long QT syndrome, splicing

## Abstract

The Kv11.1 voltage-gated potassium channel, encoded by the KCNH2 gene, conducts the rapidly activating delayed rectifier current in the heart. KCNH2 pre-mRNA undergoes alternative polyadenylation to generate two C-terminal Kv11.1 isoforms in the heart. Utilization of a poly(A) signal in exon 15 produces the full-length, functional Kv11.1a isoform, while intron 9 polyadenylation generates the C-terminally truncated, nonfunctional Kv11.1a-USO isoform. The relative expression of Kv11.1a and Kv11.1a-USO isoforms plays an important role in the regulation of Kv11.1 channel function. In this study, we tested the hypothesis that the RNA polyadenylate binding protein nuclear 1 (PABPN1) interacts with a unique 22 nt adenosine stretch adjacent to the intron 9 poly(A) signal and regulates KCNH2 pre-mRNA alternative polyadenylation and the relative expression of Kv11.1a C-terminal isoforms. We showed that PABPN1 inhibited intron 9 poly(A) activity using luciferase reporter assays, tandem poly(A) reporter assays, and RNA pulldown assays. We also showed that PABPN1 increased the relative expression level of the functional Kv11.1a isoform using RNase protection assays, immunoblot analyses, and patch clamp recordings. Our present findings suggest a novel role for the RNA-binding protein PABPN1 in the regulation of functional and nonfunctional Kv11.1 isoform expression.

## 1. Introduction

The Kv11.1 voltage-gated potassium channel conducts the rapidly activating delayed rectifier current (*I*_Kr_) in the heart and contributes to the repolarization of the cardiac action potential [1,2,3]. Kv11.1 is encoded by the KCNH2 gene or human ether-a-go-go-related gene 1 (hERG1) [4]. Mutations in KCNH2 cause long QT syndrome type 2 (LQT2), a disorder that can cause severe ventricular arrhythmias and sudden death [5,6]. KCNH2 pre-mRNA undergoes alternative processing to generate two C-terminal isoforms with distinct functional properties [7]. The Kv11.1a isoform represents the full-length functional channel and is generated following the splicing of intron 9 and polyadenylation in exon 15. The Kv11.1a-USO isoform is produced following alternative polyadenylation in intron 9 and lacks 359 C-terminal amino acids present in the Kv11.1a isoform. Kv11.1a-USO isoforms do not form functional channels when expressed in mammalian cells [7,8,9]. Because formation of Kv11.1 C-terminal isoforms from KCNH2 pre-mRNA is mutually exclusive, the nonfunctional Kv11.1a-USO isoform is generated at the expense of the functional Kv11.1a isoform. In the human heart, only one-third of KCNH2 pre-mRNA is processed to the functional Kv11.1a isoform [7]. Therefore, alternative polyadenylation is an important mechanism that regulates the relative expression levels of functional and nonfunctional Kv11.1 channels.

The alternative processing of KCNH2 pre-mRNA is directed by the competition between intron 9 splicing and intron 9 polyadenylation [7]. The competition between splicing and polyadenylation is modulated in part by several unique cis-acting elements in intron 9. These include a weak noncanonical poly(A) signal, AGUAAA, located 10−30 nt upstream of the cleavage site and U-/GU-rich downstream elements (DSE), located <30 nt downstream of the cleavage site. Replacing the weak intron 9 poly(A) signal with the strong canonical poly(A) signal, AAUAAA, leads to the predominant expression of Kv11.1a-USO [7]. The RNA-binding proteins Hu antigen R (HuR) and Hu antigen D (HuD) were recently shown to interact with the intron 9 DSE to inhibit alternative intronic polyadenylation and increase the relative expression level of the functional Kv11.1a isoform [10]. The importance of understanding the molecular mechanisms that regulate KCNH2 pre-mRNA alternative polyadenylation is highlighted by the identification of the LQT2 mutation IVS9–2delA that disrupts intron 9 splicing, leading to the predominant expression of the nonfunctional Kv11.1a-USO isoform [11].

Polyadenylate binding protein nuclear 1 (PABPN1) is a ubiquitously expressed RNA binding protein that binds to the poly(A) tail of eukaryotic mRNAs in the nucleus and plays several important roles in pre-mRNA processing. PABPN1 has been shown to recruit poly(A) polymerase to the 3′ end of nascent transcripts and increase the efficiency of polyadenylation [12]. PABPN1 restricts the length of the poly(A) tail to ~250 nt [13] and assists in the export of mRNA from the nucleus to the cytoplasm [14]. PABPN1 has also been shown to inhibit noncanonical poly(A) signals in the 3′ UTR and within intronic regions, thereby regulating the expression of transcripts with different 3′ lengths and coding sequences [15]. A unique feature of intron 9 in KCNH2 is the presence of 22 consecutive adenosine nucleotides immediately downstream of the poly(A) signal [7]. This polyadenosine stretch in KCNH2 pre-mRNA may serve as a binding site for PABPN1 prior to cleavage and addition of a poly(A) tail. In the present study, we tested the hypothesis that PABPN1 can bind the polyadenosine stretch in KCNH2 intron 9 and modulate the alternative polyadenylation of KCNH2 pre-mRNA. Our findings suggest that PABPN1 inhibits KCNH2 intron 9 polyadenylation and increases Kv11.1a isoform expression and Kv11.1 current. Our results suggest a novel role for PABPN1 in the regulation of KCNH2 alternative polyadenylation.

## 2. Results

### 2.1. Modulation of KCNH2 Intron 9 Alternative Processing by PABPN1 Using a Luciferase Reporter Construct

**Figure 1:**
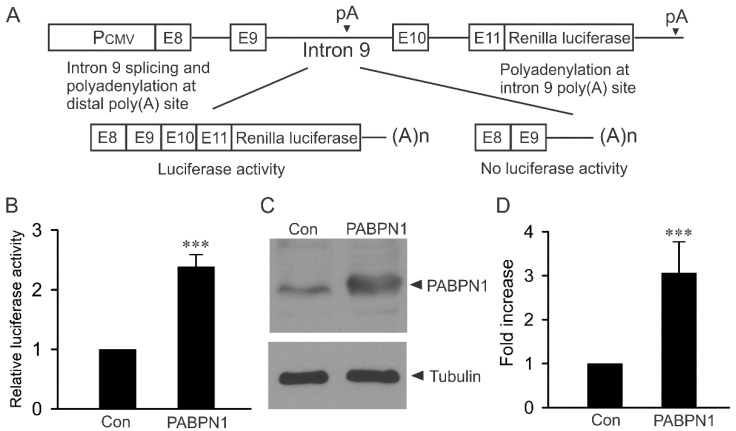
PABPN1 modulates KCNH2 intron 9 alternative processing in luciferase reporter assay. (**A**) Diagram of the KCNH2 minigene luciferase reporter construct. (**B**) Histogram showing luciferase activity was significantly increased following the co-transfection of PABPN1 compared with vector-transfected control. Data were normalized and presented as mean ± standard error (***, *p* < 0.001, n = 4). (**C**) Immunoblot analysis of PABPN1 expression from HEK293 cells following transient transfection with vector control or PABPN1 using an anti-PABPN1 antibody. (**D**) Histogram showing PABPN1 expression was significantly increased following transient transfection compared with vector-transfected control (***, *p* < 0.001, n = 4, error bars, mean ± S.E.).

To determine whether PABPN1 regulates the alternative processing of KCNH2 intron 9, we performed a luciferase reporter assay using a construct containing KCNH2 genomic DNA from exon 8 to exon 11 upstream of the *Renilla* luciferase gene (Figure 1A). In this splicing-competent minigene reporter construct, there is competition between intron 9 splicing and intron 9 polyadenylation. The splicing of intron 9 results in luciferase expression and activity, whereas the polyadenylation of intron 9 results in no luciferase expression and activity. The co-transfection HEK293 cells with PABPN1 and the luciferase reporter construct resulted in a significant increase in luciferase activity (Figure 1B). Immunoblot analysis using a PABPN1-specific antibody showed a significant increase in the expression of PABPN1 protein following transient transfection compared to endogenous levels of PABPN1 expressed from vector-transfected HEK293 cells (Figure 1C,D). This result suggests that PABPN1 can regulate alternative processing of KCNH2 intron 9, leading to an increase in luciferase activity.

### 2.2. PABPN1 Suppresses Noncanonical KCNH2 Intron 9 Poly(A) Signal Activity

**Figure 2:**
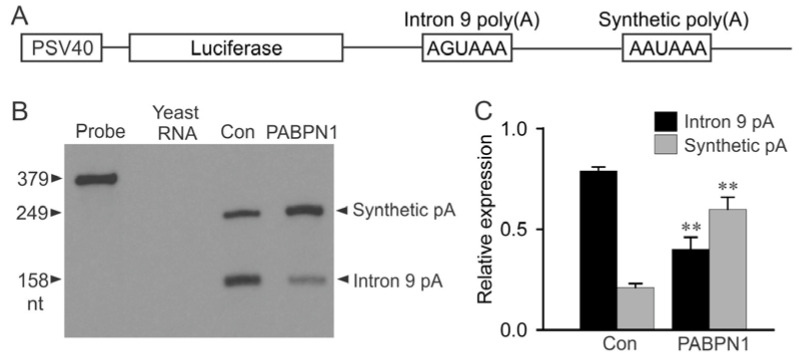
Suppression of the KCNH2 intron 9 poly(A) signal by PABPN1. (**A**) Diagram of the tandem poly(A) signal construct including the SV40 promoter, firefly luciferase gene, KCNH2 intron 9 poly(A) signal and the strong, synthetic poly(A) signal. (**B**) RNase protection assay (RPA) analysis of the relative usage of intron 9 poly(A) signal and synthetic poly(A) signal following the co-transfection of HEK293 cells with the tandem poly(A) signal construct and vector control (Con) or with PABPN1. Yeast RNA was used as a control for the complete digestion of the probes by RNase. (**C**) Histogram plotted as the expression of intron 9 poly(A) and synthetic poly(A) relative to the total intron 9 poly(A) + synthetic poly(A) signal. The activity of the intron 9 poly(A) signal significantly decreased following co-transfection with PABPN1 compared with vector-transfected control (black bars) and the activity of the synthetic poly(A) signal significantly increased following co-transfection with PABPN1 compared with vector-transfected control (gray bars) (**, *p* < 0.01, n = 3, mean ± S.E.).

The PABPN1-mediated increase in luciferase activity may result from the inhibition of intron 9 polyadenylation or from the enhancement of intron 9 splicing. To test whether the increase in luciferase activity is due to the inhibition of KCNH2 intron 9 poly(A) signal activity, we performed a competition assay using a tandem poly(A) signal construct containing the *Renilla* luciferase gene positioned upstream of a 302 nt segment of KCNH2 intron 9 that included the noncanonical poly(A) signal and 22 nt adenosine stretch followed by a strong, synthetic poly(A) signal (Figure 2A). The RNA generated from the tandem poly(A) signal construct was analyzed by the RNase protection assay (RPA) using a probe specific to KCNH2 intron 9. If the intron 9 poly(A) signal is used, a 158 nt fragment is generated following RNase digestion, while a 249 nt RNA fragment is generated following utilization of the synthetic poly(A) signal. When the tandem poly(A) construct was co-transfected with vector control, the 158 nt fragment was predominantly generated, indicating that transcription was terminated at the proximal intron 9 poly(A) signal (Figure 2B). The co-transfection of HEK293 cells with PABPN1 and the tandem poly(A) signal construct resulted in a significant decrease in utilization of the intron 9 poly(A) signal from 79% to 40%, and a concomitant increase in the usage of the synthetic poly(A) signal from 21% to 60% (*p* < 0.01, n = 3) (Figure 2C). These results suggest that the KCNH2 intron 9 poly(A) signal activity is inhibited by PABPN1.

### 2.3. Interaction between PABPN1 and KCNH2 Intron 9 Poly(A) Signal and Polyadenosine Stretch

**Figure 3:**
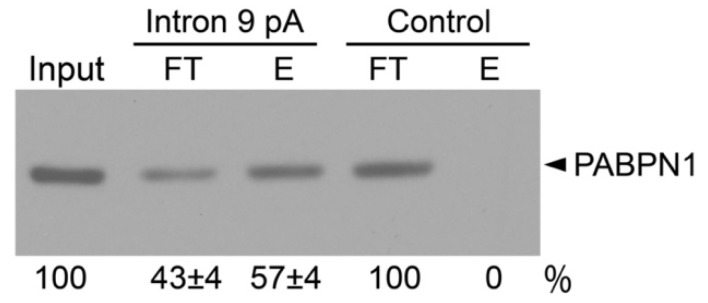
Interaction between PABPN1 and KCNH2 intron 9. Cell lysates from HEK293 cells were incubated with biotinylated and streptavidin bead–bound RNA probes corresponding to the KCNH2 intron 9 poly(A) signal and polyadenosine stretch or the negative control oligo containing the sequence from the 3′ UTR of androgen receptor mRNA. The RNA-bound protein fractions (E) and unbound fractions (FT) were analyzed by immunoblotting with anti-PABPN1 antibody. Signals were quantified and expressed as percentage of total (FT + E) protein (n = 3, mean ± S.E.).

PABPN1 has been reported to minimally bind to sequences containing 10-12 consecutive adenosine nucleotides [16]. To determine whether PABPN1 physically interacts with the polyadenosine stretch within KCNH2 intron 9, we performed a biotinylated RNA pulldown assay. HEK293 cell lysates were incubated with a biotinylated RNA oligo comprising the intron 9 poly(A) signal and polyadenosine stretch. Immunoblot analysis with a PABPN1-specific antibody showed that the protein level in the streptavidin-retained fraction was greater than the protein level in the unbound fraction (Figure 3). PABPN1 had no interaction with the negative control oligo containing the sequence from the 3′ UTR of androgen receptor mRNA. These results support the hypothesis that PABPN1 binds to the 22 nt adenosine stretch within KCNH2 intron 9 and inhibits intron 9 polyadenylation.

### 2.4. Regulation of Kv11.1 Isoform Expression by PABPN1

**Figure 4:**
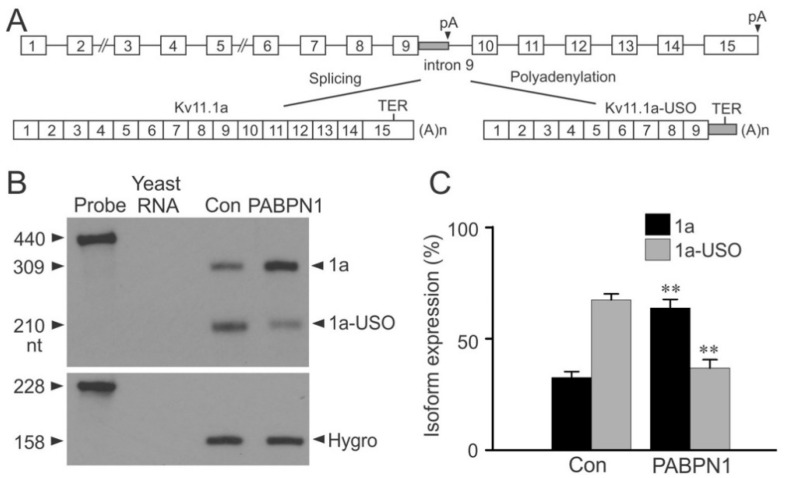
Regulation of Kv11.1 isoform expression by PABPN1. (**A**) Diagram of the short KCNH2 gene construct and the generation of the Kv11.1 C-terminal isoforms. (**B**) RPA analysis of mRNA from Flp-In HEK293 cells stably expressing the short KCNH2 gene following transfection with vector control or PABPN1. Yeast RNA was used as a control for the complete digestion of the probes by RNase. (**C**) The RPA signals were quantified, normalized to the expression of the hygromycin B resistance gene (Hygro) and plotted as the relative expression of the total isoform (Kv11.1a + Kv11.1a-USO) expression. Co-transfection with PAPBN1 significantly increased the relative expression of Kv11.1a transcripts (black bars) and significantly decreased expression of Kv11.1a-USO transcripts (gray bars) compared with vector-transfected control (**, *p* < 0.01, n = 3, error bars, mean ± S.E.).

To test whether the inhibition of the KCNH2 intron 9 poly(A) signal regulated the relative expression of Kv11.1 isoforms, we performed RPA analysis using a splicing-competent short KCNH2 gene construct. The generation of the short KCNH2 gene construct has been previously described [11]. In HEK293 cells, the short KCNH2 gene undergoes alternative processing and generates Kv11.1a and Kv11.1a-USO transcripts (Figure 4A). We transiently transfected PABPN1 into Flp-In HEK293 cells that stably express the short KCNH2 gene. RNA was analyzed by RPA using a probe specific to KCNH2 exon 9 and exon 10. If intron 9 is removed by splicing, a 309 nt fragment is protected following RNase digestion. Alternatively, a 210 nt RNA fragment is generated following utilization of the KCNH2 intron 9 poly(A) signal (Figure 4B). RPA analysis showed that transfection of PABPN1 resulted in a significant increase in the expression of the Kv11.1a transcript and a significant decrease in the expression of the Kv11.1a-USO transcript (Figure 4C). This result indicates that relative expression of Kv11.1 C-terminal isoforms can be regulated by PABPN1.

### 2.5. Upregulation of Kv11.1a Isoform Protein Expression by PABPN1

**Figure 5:**
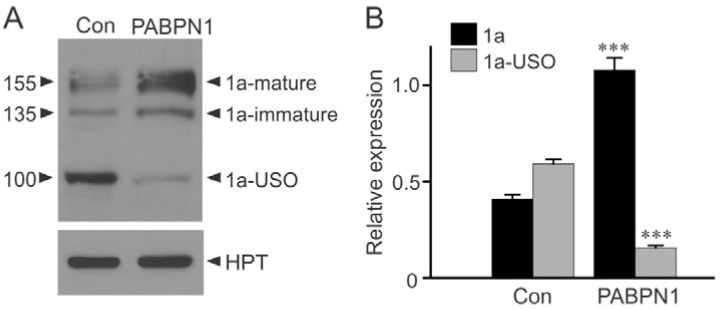
Regulation of Kv11.1 isoform protein expression by PABPN1. (**A**) Immunoblot analysis of Kv11.1 protein from Flp-In HEK293 cells stably expressing the short KCNH2 gene following transfection of vector control (Con) or PABPN1. The expression level of hygromycin B phosphotransferase (HPT) served as a loading control. Cell lysates were subjected to SDS-PAGE and probed with antibodies against the N-terminus of Kv11.1 or to HPT. (**B**) Histogram showing significantly increased expression of the Kv11.1a isoform (1a-mature and 1a-immature, black bars) and significantly decreased expression of Kv11.1a-USO (gray bars) following transfection with PABPN1 compared with vector-transfected control (***, *p* < 0.001, n = 3, error bars, mean ± S.E.). The protein bands were quantified, normalized to HPT, and plotted as relative expression of total Kv11.1 protein (1a + 1a-USO) in vector control.

To determine whether the inhibition of the KCNH2 intron 9 poly(A) signal could increase the expression of the full-length Kv11.1a isoform protein we analyzed relative expression of Kv11.1 isoforms using immunoblot analyses. PABPN1 was transiently transfected into Flp-In HEK293 cells stably expressing the short KCNH2 gene construct. We used an antibody that recognizes both Kv11.1a and Kv11.1a-USO isoforms expressed from the short KCNH2 gene construct. The Kv11.1a isoform is expressed as two protein bands at 155 and 135 kDa, representing the complex-glycosylated mature channel protein and core-glycosylated immature form of the protein, respectively. A third protein band at 100 kDa represents the truncated Kv11.1a-USO isoform (Figure 5A). We observed a significant increase in Kv11.1a protein levels and a significant decrease in the expression of Kv11.1a-USO protein levels following PABPN1 transfection (Figure 5A,B).

### 2.6. PABPN1 Increases Kv11.1 Channel Current

**Figure 6:**
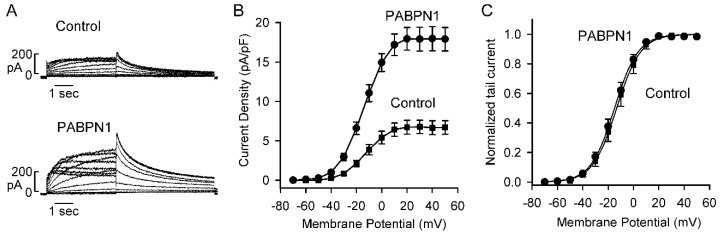
Effect of PABPN1 on Kv11.1 channel current. (**A**) Representative currents recorded from Flp-In HEK293 cells stably expressing the short KCNH2 gene following transfection with GFP vector (Control) or PABPN1+GFP. (**B**) I-V plot of tail current density measured at −50 mV following test voltages from −70 to +50 mV for vector-transfected control (squares, n = 10) or PABPN1 (circles, n = 7, error bars, mean ± S.E.). (**C**) Activation curves measured with normalized tail currents and fitted to a Boltzmann equation for vector-transfected control (squares, n = 10) and PABPN1 (circles, n = 7, error bars, mean ± S.E.).

To study whether PABPN1 modulates the functional properties of Kv11.1 channels expressed from the short KCNH2 gene, we performed patch clamp experiments. HEK293 cells stably expressing the short KCNH2 gene were transiently transfected with control or PABPN1 plasmid. Kv11.1 current was activated by depolarizing steps between −70 and +50 mV from a holding potential of −80 mV. Kv11.1 tail current was recorded following repolarization to −50 mV. We observed a significant increase in Kv11.1 current following PABPN1 transfection (Figure 6A,B). The Kv11.1 current amplitude in the vector control represents the current expressed from approximately one third of the KCNH2 pre-mRNA being processed to the full-length functional isoform. The voltage dependence of Kv11.1 channel activation was determined by fitting the normalized tail currents with a Boltzmann function (Figure 6C). The half maximal activation voltages (V_1/2_) for control and PABPN1 were –13.2 ± 2.8 mV and –15.0 ± 2.0 mV, respectively. The increased current is consistent with RPA and immunoblot results showing that PABPN1 increases the functional Kv11.1a isoform and decreases the nonfunctional Kv11.1a-USO isoform.

## 3. Discussion

In the present study, we have shown that the RNA polyadenylate binding protein nuclear 1 modulates the relative expression of Kv11.1 C-terminal isoforms by suppressing KCNH2 intron 9 poly(A) signal activity and increasing the expression level of the full-length functional Kv11.1a isoform. The inhibition of alternative polyadenylation of KCNH2 pre-mRNA by PABPN1 was characterized using KCNH2 minigene reporter assays and RNA pulldown assays. The increase in the relative expression of the Kv11.1a isoform was determined at the RNA, protein, and functional levels using short KCNH2 gene constructs in RPA, immunoblot and patch clamp experiments. Our results identify KCNH2 intron 9 as a novel target for PABPN1 and provides new insights into the role that alternative polyadenylation plays in the post-transcriptional regulation of Kv11.1 isoform expression.

The processing of pre-mRNA in the nucleus is an essential regulatory mechanism that guides the spatiotemporal patterns of eukaryotic gene expression. Alternative polyadenylation represents an important post-transcriptional mechanism regulating gene expression, with approximately 80% of mammalian genes containing more than one poly(A) signal [17]. Alternative poly(A) site usage can lead to the production of different protein isoforms or the production of different transcripts encoding the same protein but with 3′ UTRs with varying lengths. PABPN1 has been shown to play a key role in mRNA processing. PABPN1 was originally identified for its role as a protein that binds to the poly(A) tail after the initial 11 to 14 adenosines have been added [18] and has been shown to stabilize the nascent transcript, increase the efficiency of poly(A) polymerase, and restrict the length of the poly(A) tails to ~250 nt in mammalian cells [13,14]. PABPN1 has also been shown to play a role in alternative polyadenylation. Jenal et al. showed that PABPN1 binds to and suppresses weak, noncanonical proximal poly(A) signals in the 3′ UTR or intronic regions of mammalian RNAs [15]. Our present results show that PABPN1 significantly increases the functional Kv11.1a isoform by suppressing KCNH2 intron 9 poly(A) signal activity. A unique feature of the KCNH2 intron 9 poly(A) signal region is the presence of a 22 nt adenosine stretch immediately downstream of noncanonical poly(A) signal, AGUAAA. Our previous studies suggest that the cleavage of KCNH2 intron 9 poly(A) site occurs within the polyadenosine stretch [7]. It is conceivable that the interaction between PABPN1 and KCNH2 intron 9 pre-mRNA may interfere with polyadenylation factors in recognition and cleavage of the KCNH2 intron 9 poly(A) site.

The competition between intron 9 splicing and polyadenylation determines the relative expression level of the Kv11.1a and Kv11.1a-USO isoforms [7]. In addition to the weak intron 9 poly(A) signal, several additional cis-acting elements have been shown to contribute to regulation of Kv11.1 isoform expression including an intrinsically weak splice donor site and U-/GU-rich DSE. We have previously shown that modified U1 snRNA with increased complementarity to the weak 5′ splice site of KNCH2 intron 9 significantly increases in the efficiency of intron 9 splicing and upregulates expression of the functional Kv11.1a isoform [19]. In addition, we found that RNA-binding proteins HuR and HuD inhibit the KCNH2 intron 9 poly(A) signal by binding to the intron 9 DSE and increase expression of the functional Kv11.1a isoform [10]. Our results identify PABPN1 as a trans-acting factor that recognizes the polyadenosine stretch within intron 9 and inhibits intron 9 poly(A) signal activity. The inhibition of KCNH2 intron 9 polyadenylation leads to increased intron 9 splicing and increased expression of the functional Kv11.1a isoform and channel current. Thus, the present work demonstrates the increasingly complex mechanisms that contribute to Kv11.1 isoform expression in which several cis-acting elements control KCNH2 intron 9 splicing and polyadenylation through interaction with different trans-acting factors.

The identification of a KCNH2 mutation that causes LQT2 by an isoform switch mechanism highlights the functional significance of Kv11.1 isoform expression [11]. The mutation, IVS9-2delA, is a single nucleotide deletion in the 3′ acceptor site of intron 9 that disrupts splicing of intron 9 and results in the exclusive expression of the Kv11.1a-USO isoform in the mutant allele. Thus, the relative expression of Kv11.1a and Kv11.1a-USO isoforms plays an important role in the regulation of Kv11.1 channel function. The role that alternative polyadenylation plays in regulating gene expression is a relatively new and exciting area of research. A recent genome-wide polyadenylation study by Creemers et al. found that alternative polyadenylation was modulated in the hearts of patients with dilated cardiomyopathy compared to healthy hearts [20]. Interestingly, the authors reported that PABPN1 was downregulated at the RNA and protein level in the failing hearts. Decreased amounts of PABPN1 has been shown to increase the usage of proximal poly(A) sites in the 3′ UTR [21,22]. The downregulation of PABPN1 has also been reported in oculopharyngeal muscular dystrophy (OPMD). OPMD is a late-onset neurodegenerative disease characterized by dysphagia and proximal limb weakness [23]. The pathological hallmark of this disease is the accumulation of insoluble aggregates of the alanine-expanded mutant PABPN1 proteins (exp-PABPN1) and depleting levels of soluble PABPN1. The expansion of the polyalanine stretch in the N-terminal end of exp-PABPN1 is due to a short expansion of the triplet repeat GCG_6_ to GCG_8-13_ in the PABPN1 gene. Genome-wide polyadenylation studies have identified widespread changes in gene expression profiles in mice expressing exp-PABPN1 including increased utilization of proximal poly(A) sites within the 3′ UTR [15] and within intronic poly(A) sites [24]. Recently, an exp-PABPN1 transgenic mouse model generated by Mankodi et al. revealed nuclear aggregates in the cardiac muscle of mice that preceded the development of cardiomyopathy [25]. In our present results, we identified a novel function of PABPN1 in suppressing KCNH2 intron 9 polyadenylation and regulating Kv11.1 isoform expression. Whether downregulation of PABPN1 observed in dilated cardiomyopathy and OPMD results in dysregulation of Kv11.1 isoform expression and leads to the development of arrhythmias requires future investigation.

## 4. Materials and Methods

### 4.1. Plasmid Constructs and Transfections

The creation of the KCNH2 minigene luciferase reporter construct has been previously described [26]. Briefly, KCNH2 genomic DNA from exon 8 to exon 11 was subcloned upstream of the *Renilla* luciferase gene. The expression level of *Renilla* luciferase depends on the competition between intron 9 splicing and intron 9 polyadenylation. The vector also contains the firefly luciferase gene, which was used as a control for transfection efficiency. HEK293 cells were transiently transfected with the KCNH2 minigene luciferase reporter construct using the Effectene method (Qiagen, Valencia, CA, USA) and *Renilla* and firefly luciferase activity was detected using the dual-luciferase assay kit (Promega, Madison, WI, USA) as previously described [10]. Data were analyzed by normalizing *Renilla* luciferase activity to firefly luciferase activity and presented as mean ± standard error.

The tandem poly(A) signal construct has been described previously [7]. Briefly, 308 bp of KCNH2 intron 9 was subcloned downstream of the firefly luciferase gene and upstream of a strong synthetic poly(A) signal. The expression of the tandem poly(A) signal construct was driven by the SV40 promoter. HEK293 cells were transiently transfected with the tandem poly(A) construct using the Effectene method.

The generation of the short KCNH2 gene construct in which the two longest introns, intron 2 (14.9 kb) and intron 5 (4.4 kb), are shortened to 600 bp has been previously described [11]. The short KCNH2 gene construct contains the hygromycin resistance gene with a Flp recombination target site, which allows stable transfection into Flp-In HEK293. PABPN1 cDNA in pcDNA3.1 plasmid was used in all PABPN1 transfection experiments except for patch clamp experiments, where the plasmid expressing both GFP and PABPN1 was used. For the PABPN1+GFP plasmid, the GFP coding sequence was subcloned into a PGL-3 promoter vector at HindIII and XbaI sites. The SV40 promoter-GFP fragment was removed at BglII and BamHI sites and subcloned into pcDNA3.1-PABPN1 plasmid at BglII site. Thus, in the pcDNA3.1-PABPN1+GFP plasmid, the expression of GFP is driven by the SV40 promoter and the expression of PABPN1 is driven by the CMV promoter.

The short KCNH2 gene construct was stably expressed in Flp-In HEK293 by co-transfection of the short KCNH2 gene (0.1 μg) with the Flp recombinase expression vector pOG44 (0.9 μg) using the Effectene method (Qiagen) and selected with 100 μg/mL hygromycin. Flp-In HEK293 cells contain the FRT site at a single genomic locus, allowing stable integration of a single copy of the short KCNH2 gene via Flp recombinase-mediated DNA recombination. The plasmids expressing PABPN1 or PABPN1+GFP were transiently transfected into the Flp-In HEK293 cells that stably express the short KCNH2 gene construct using PolyJet transfection reagent. Flp-In HEK293 cells were cultured in DMEM supplemented with 10% FBS. For electrophysiological studies, the cells were harvested from the culture dish by trypsinization, washed twice with standard MEM medium, and stored in this medium at room temperature for later use.

### 4.2. Biotinylated RNA Pulldown Assay

The biotinylated RNA pull-down assay was performed using a Magnetic RNA-Protein Pull-Down Kit (Thermo Scientific, Rockford, IL, USA) as described previously [10]. RNA oligos containing KCNH2 intron 9 poly(A) signal AGUAAA and 22 nt adenosine stretch were custom synthesized by Genscript (Piscataway, NJ, USA). An RNA oligo containing a sequence in the 3′ UTR of androgen receptor mRNA was used as a negative control. The RNA oligos were labeled with biotin using RNA 3′ End Desthiobiotinylation Kit (Thermo Scientific). The biotinylated RNAs were extracted with chloroform:isoamyl alcohol, precipitated with ethanol, rehydrated in nuclease-free water and bound to streptavidin magnetic beads. The cell lysates were prepared by mammalian protein extraction reagent (M-PER) (Thermo Scientific). Briefly, PABPN1/RNA complexes were allowed to form at 4 °C for 60 min in 50 µL mixtures containing 50 pmole biotinylated RNA probe, 50 µg of cell lysate in 1X binding buffer with 15% glycerol. PABPN1/RNA complexes were washed with 50 µL of 1X wash buffer twice, and then bead-associated proteins were eluted with 50 µL of elution buffer for 30 min at 37 °C. The eluted samples were heated for 5 min at 95 °C in the presence of SDS–PAGE loading buffer, and then analyzed by immunoblot.

### 4.3. RNase Protection Assay

RNA isolation and the RNase protection assay were performed as previously described [11]. Briefly, RNA isolated from Flp-In HEK293 cells stably expressing the short KCNH2 gene were analyzed with the riboprobes using the RPAII and BrightStart BioDetect kits (Ambion, Austin, TX, USA). Antisense RNA riboprobes were transcribed in vitro in the presence of biotin-14-CTP. The expression of RNA transcribed from the hygromycin B resistance gene in the short KCNH2 gene construct was used as an internal control for normalization of the expression of Kv11.1 isoform transcripts. The probe used to detect the RNA contained 158 nt of the hygromycin B resistance gene and 70 nt from the pGEM vector. Yeast RNA was used as a control for the complete digestion of the probes by RNase. The intensity of each band was quantified using Image J software and adjusted for the number of biotin-labeled cytidines in each protected fragment.

### 4.4. Immunoblot Analysis

Immunoblot analysis was performed as previously described [7]. Kv11.1a and Kv11.1a-USO proteins were detected using an anti-Kv11.1 antibody directed against the N-terminus of the isoforms (H-175, Santa Cruz, CA, USA). The relative expression levels of Kv11.1a and Kv11.1a-USO were normalized to the expression of hygromycin B phosphotransferase (HPT) encoded by the hygromycin B resistance gene [7]. PABPN1 was detected using an anti-PABPN1 antibody (Abcam, Cambridge, MA, USA). The expression of β-tubulin was used as a loading control as previously described [10]. The intensity of each protein band was quantified using Image J software.

### 4.5. Patch Clamp Recordings

The recording of membrane currents in the whole cell configuration has been previously described [3,27]. Cells were superfused with HEPES-buffered solution containing 1.8 mM CaCl_2_, 4 mM KCl, 1 mM MgCl_2_, 137 mM NaCl, 10 mM HEPES and 10 mM glucose (pH 7.4). The recording pipettes were fabricated from Kimax borosilicate glass tubes using a P-97 microelectrode puller (Sutter Instrument, Novato, CA, USA). The pipettes had an inner diameter of 1–1.5 µm, and when filled with the internal pipette solution, had a resistance of 2–4 MΩ. The internal pipette solution contained 130 mM KCl, 1 mM MgCl_2_, 5 mM MgATP, 10 mM HEPES and 5 mM EGTA (pH 7.2). An Axopatch-200B patch-clamp amplifier and pCLAMP10 software (Molecular Devices, Sunnyvale, CA, USA) were used to record membrane currents. The data were low pass-filtered at 2 kHz and digitalization at 5 kHz. Kv11.1 current was activated by depolarizing steps between −70 and +50 mV from a holding potential of −80 mV. Kv11.1 tail current was recorded following repolarization to −50 mV. Activation curves were generated by fitting normalized tail currents with a Boltzmann distribution: I(V)/I_max_ = 1/[1 + exp((V_1/2_ − V)/k)], where V is the depolarizing pulse potential, V_1/2_ is the membrane potential at half-activation, and k is the slope factor. All patch clamp experiments were performed at room temperature. According to Conforti et al., pipette solutions with KCl and extracellular solutions with NaCl may result in a small junction potential (2–3 mV) [28], and we did not correct for this.

### 4.6. Data Analysis

Data are presented as mean ± standard error (S.E.) and analyzed by Student’s *t*-test using SigmaPlot (San Jose, CA, USA). *p* values < 0.05 are considered statistically significant.

## Data Availability

Data sharing not applicable.

## Abbreviations

KCNH2	Potassium voltage-gated channel subfamily H member 2
PABPN1	Polyadenylate binding protein nuclear 1
LQT2	Long QT syndrome type 2
nt	Nucleotide
RPA	RNase protection assay
DSE	Downstream sequence elements
HPT	Hygromycin B phosphotransferase
Hygro	Hygromycin B resistance gene
